# Severe Maternal Morbidity by Race and Ethnicity and Birth Mode Among Individuals With a Prior Cesarean Birth

**DOI:** 10.1001/jamanetworkopen.2025.13578

**Published:** 2025-06-03

**Authors:** Laura B. Attanasio, Sarah Goff, Rachel Hardeman, Holly Laws, Sindhu Srinivas

**Affiliations:** 1 Department of Health Promotion and Policy, University of Massachusetts Amherst, Amherst; 2 Division of Health Policy and Management, University of Minnesota School of Public Health, Minneapolis; 3 Center for Research on Families, University of Massachusetts Amherst, Amherst; 4 Department of Obstetrics and Gynecology, University of Pennsylvania Perelman School of Medicine, Philadelphia

## Abstract

**Importance:**

Given that nearly one-third of US births are cesarean deliveries, subsequent births after a cesarean delivery are common. Racial and ethnic disparities in severe maternal morbidity (SMM) have been well-documented, and prior studies have identified differences in birth mode after prior cesarean delivery by race and ethnicity.

**Objective:**

To examine variation by race and ethnicity in the association between SMM and birth mode for individuals with a prior cesarean delivery.

**Design, Setting, and Participants:**

This cross-sectional study used Massachusetts linked birth certificate and hospital discharge data from 2012 to 2021. The analytic sample was limited to births to individuals with 1 prior cesarean delivery. Data were analyzed from August 23, 2024, to March 31, 2025.

**Exposures:**

Race and ethnicity and birth mode (vaginal birth after cesarean delivery, planned repeat cesarean delivery, and unplanned repeat cesarean delivery).

**Main Outcome and Measures:**

SMM was measured using Centers for Disease Control and Prevention indicators. Associations of race and ethnicity and birth mode with SMM were calculated using logistic regression, then an interaction term was added between race and ethnicity and birth mode. Models controlled for covariates.

**Results:**

The study population included 72 836 individuals (mean [SD] age, 32.40, [5.03] years), of whom 8022 (11.0%) were Black, 14 664 (20.1%) were Latinx, and 41 350 (56.8%) were White. Approximately one-third of individuals were born outside the US (25 119 individuals [34.5%]). In adjusted analyses, Black individuals had higher odds of SMM compared with White individuals (adjusted odds ratio [AOR], 1.60; 95% CI, 1.25-2.05). Odds of SMM were higher for unplanned repeat cesarean birth (AOR, 3.05; 95% CI, 2.23-4.18) compared with vaginal birth after cesarean delivery, and higher for planned repeat cesarean birth compared with vaginal birth after cesarean delivery (AOR, 1.57; 95% CI, 1.20-2.06). Including an interaction term identified variation in the association between birth mode and SMM by race and ethnicity. Planned repeat cesarean birth vs VBAC was associated with an increase in the likelihood of SMM of 0.56 (95% CI, 0.21-0.90) percentage points (*P* = .001) among Black birthing people and 0.46 (95% CI, 0.16-0.76) percentage points (*P* = .003) among Latinx birthing people, while among White individuals, the likelihood of SMM did not differ between planned repeat cesarean birth and VBAC.

**Conclusions and Relevance:**

In this cross-sectional study of births among individuals with a prior cesarean birth, patterns of SMM by birth mode varied by race and ethnicity, with elevated rates of SMM among those from marginalized racial and ethnic groups with planned cesarean births. Future work should identify interventions to improve quality of care and promote equity for this population.

## Key Points

**Question:**

Does the association between birth mode and severe maternal morbidity (SMM) among those with a prior cesarean birth vary by race and ethnicity?

**Findings:**

In this cross-sectional study of 72 836 individuals with a prior cesarean delivery, SMM rates by birth mode differed by race and ethnicity. Black and Latinx individuals experienced higher SMM rates with planned repeat cesarean vs vaginal birth after cesarean birth, while among White individuals, SMM was similar for planned repeat cesarean birth and vaginal birth after cesarean birth.

**Meaning:**

Differential patterns of SMM by race and ethnicity and birth mode among individuals with prior cesarean indicate a need to identify factors contributing to higher SMM rates among Black and Latinx individuals with planned repeat cesarean delivery.

## Teaser

This cross-sectional study examines associations of race and ethnicity with severe maternal morbidity and birth mode for individuals with history of cesarean delivery.

## Introduction

Severe maternal morbidity (SMM) impacts more than 50 000 birthing individuals each year in the US and has increased in recent decades.^1,2^ Nationally, rates of SMM are 1.6 times higher for Black birthing people and 1.2 times higher for Latinx birthing people than for White birthing people,^3^ with wider disparities in some geographic areas.^4^ Racial and ethnic disparities in SMM persist after stratifying by insurance type and socioeconomic status.^5,6^ Comorbidities that increase SMM risk are more common among Black individuals compared with White individuals, but do not account for SMM disparities.^7^ SMM rates are higher among those with cesarean birth vs vaginal birth.^2,8^

Black birthing people have higher rates of primary cesarean birth compared with White birthing people,^9,10,11^ resulting in disproportionate representation of Black individuals among those with a prior cesarean birth. Individuals with a prior cesarean birth can have a planned repeat cesarean birth or labor after cesarean delivery (LAC), with the goal of having a vaginal birth after cesarean delivery (VBAC). Maternal morbidity is lowest for those who have VBAC and lower for planned repeat cesarean birth than for unplanned cesarean delivery.^12,13^ Among birthing people with a prior cesarean birth, Black individuals are more likely to have LAC than White individuals, but have lower rates of VBAC following LAC, even after controlling for comorbidities.^14,15^ Due to the different patterns of LAC and VBAC by race and ethnicity, Black birthing people are more likely to have unplanned cesarean delivery compared with White birthing people, potentially contributing to racial and ethnic disparities in SMM.

Racial and ethnic disparities in SMM have been well-documented, and prior studies have identified racial and ethnic differences in birth mode after prior cesarean delivery, but the intersection of race and ethnicity, birth mode, and SMM among individuals with a prior cesarean delivery has not been examined. The goal of this study was to characterize the association between birth mode and SMM among individuals with a prior cesarean delivery and to examine whether this association varies by race and ethnicity.

## Methods

### Data and Sample

This cross-sectional study was reviewed and approved by institutional review boards of the University of Massachusetts Amherst and Massachusetts Department of Public Health. This study was exempt from the requirement of informed consent due to the use of preexisting secondary data. Data are from the Massachusetts Pregnancy to Early Life Longitudinal Data System for 2012 to 2021, which contains birth certificate data linked to hospital discharge records. This study adhered to the Strengthening the Reporting of Observational Studies in Epidemiology (STROBE) reporting guideline.

The study sample included individuals aged 18 years or older with a singleton live birth at 34 to 42 weeks’ gestation and 1 prior cesarean delivery. We excluded birthing people with a clear contraindication to vaginal delivery or whose newborn had a congenital anomaly.^16^ Due to the *International Classification of Diseases, Ninth Revision (ICD-9)* to *International Statistical Classification of Diseases and Related Health Problems, Tenth Revision *(*ICD-10*) transition, we excluded births from the fourth quarter of 2015.^17^ Exclusions and construction of the analytic sample are shown in eFigure 1 in Supplement 1.

### Measures

The dependent variable was SMM. We constructed a binary variable for this composite measure based on the Center for Disease Control and Prevention’s published list of *ICD-9* and *ICD-10* codes indicating delivery hospitalizations with SMM.^18^ Following recent literature on SMM measurement, our primary outcome measure excluded individuals for whom blood transfusion was the only SMM indicator.^19,20,21^ In a sensitivity analysis, we used a version of the SMM measure including blood transfusion.

Key independent variables included LAC and birth mode after prior cesarean birth. Whether LAC occurred was determined based on a combination of variables from the birth certificate and diagnosis and procedure codes in hospital discharge records that suggest that labor occurred, based on published methods for identifying LAC in linked birth certificate and hospital discharge data.^22^ A list of codes we included is provided in eTable 1 in Supplement 1. Birth mode after prior cesarean birth included planned repeat cesarean birth, vaginal birth after cesarean birth, and unplanned repeat cesarean birth. Planned repeat cesarean birth was defined as a cesarean birth that occurred without labor, while cesarean births following labor were classified as unplanned repeat cesarean births.

Another key independent variable was maternal race and ethnicity, a proxy for exposure to structural and interpersonal racism. Race is a social construct grouping people based on perceived physical attributes, while ethnicity refers to a group of people with a shared cultural background.^23^ These concepts are complex and overlapping and can be problematic to measure with separate variables, given that in the US, many people who identify as Hispanic and/or Latino/Latina/Latinx ethnicity do not also identify with a racial group.^24,25^ For this reason, and consistent with much of the literature in this area, we constructed a single variable of race and ethnicity based on the data from the birth certificate, which is self-reported by birthing parents.^26^ This variable included categories of Black, not Hispanic or Latino/Latina/Latinx; Hispanic or Latino/Latina/Latinx; White, not Hispanic or Latino/Latina/Latinx; and other race, not Hispanic or Latino/Latina/Latinx (eg, American Indian or Alaska Native, Asian, Pacific Islander, and unreported race). For brevity, we refer to these categories hereafter as Black, Latinx, White, and other race or ethnicity, respectively.

Covariates included age category, education level, insurance type, born in the US (yes or no), gestational age category, parity, prepregnancy body mass index (BMI; calculated as weight in kilograms divided by height in meters squared) category, and year in which the birth took place. We calculated an obstetric comorbidity score,^27^ which adjusts for a range of conditions associated with higher SMM risk. The score was calculated separately for SMM excluding transfusion (main analysis) and SMM including transfusion (sensitivity analysis). Higher scores indicate higher risk for SMM based on comorbidities. We present descriptive statistics for chronic hypertension, preeclampsia, gestational diabetes, and preexisting diabetes. However, these conditions are included in the obstetric comorbidity index and we did not control for them separately in multivariate models.

### Statistical Analysis

We examined frequencies and bivariate associations between birth mode, race and ethnicity, and SMM. Next, we estimated logistic regression models with SMM as the outcome and birth mode and race and ethnicity as key independent variables, controlling for covariates. Finally, we added an interaction term between birth mode and race and ethnicity to the model to determine whether race and ethnicity moderates the association between birth mode and SMM, calculating average marginal effects and estimated probabilities to make the results interpretable. We then repeated this analysis using the binary LAC variable rather than 3-category birth mode. Standard errors were clustered at the hospital level. *P* values were 2-sided, and statistical significance was set at *P* ≤ .05. All analyses were conducted in Stata software version 18 (Stata Corp). Data were analyzed from August 23, 2024, to March 31, 2025.

## Results

The analytic sample included 72 836 birthing people (mean [SD] age, 32.40, [5.03] years) (Table 1). There were 8022 Black individuals (11.0%), 14 664 Latinx individuals (20.1%), 41 350 White individuals (56.8%), and 8800 individuals (12.1%) who identified as another race or ethnicity. The largest proportion of birthing people were aged 30 to 34 years (27 423 individuals [37.7%]), while 20 809 individuals (28.6%) were aged 35 to 39 years and 14 133 individuals (19.4%) were aged 25 to 29 years. Approximately one-third were born outside the US (25 119 individuals [34.5%]). Nearly half of individuals had a college degree or higher (34 805 individuals [47.8%]), and half had private insurance (36 223 individuals [49.7%]). More than two-thirds of the sample had a planned repeat cesarean birth (50 276 individuals [69.0%]), while 22 495 individuals (30.9%) had LAC. Overall, 14 828 individuals (20.4%) had VBAC and 7732 individuals (10.6%) had an unplanned repeat cesarean delivery. A total of 553 individuals (0.8%) experienced SMM. When blood transfusion was included in the definition of SMM, 1352 individuals (1.9%) had SMM.

**Table 1.** Characteristics of Study Sample by Birth Mode Among Individuals With a Prior Cesarean Birth, Massachusetts, 2012-2021

Characteristic	Individuals, No. (%)				*P* value
	Total (N = 72 836)	VBAC (n = 14 828)	Unplanned cesarean (n = 7732)	Planned cesarean (n = 50 276)	
LAC					
No	50 341 (69.1)	65 (0.4)	0	50 276 (100)	<.001
Yes	22 495 (30.9)	14 763 (99.6)	7732 (100)	0	
Race and ethnicity					
Black	8022 (11.0)	1869 (12.6)	1229 (15.9)	4924 (9.8)	<.001
Hispanic or Latinx	14 664 (20.1)	3223 (21.7)	1687 (21.8)	9754 (19.4)	
White	41 350 (56.8)	7614 (51.3)	3763 (48.7)	29 973 (59.6)	
Other^a^	8800 (12.1)	2122 (14.3)	1053 (13.6)	5625 (11.2)	
Year					
2012	7782 (10.7)	1317 (8.9)	550 (7.1)	5915 (11.8)	<.001
2013	7415 (10.2)	1401 (9.4)	580 (7.5)	5434 (10.8)	
2014	7604 (10.4)	1484 (10.0)	634 (8.2)	5486 (10.9)	
2015	5802 (8.0)	1073 (7.2)	526 (6.8)	4203 (8.4)	
2016	7614 (10.5)	1608 (10.8)	865 (11.2)	5141 (10.2)	
2017	7620 (10.5)	1577 (10.6)	974 (12.6)	5069 (10.1)	
2018	7219 (9.9)	1553 (10.5)	880 (11.4)	4786 (9.5)	
2019	7332 (10.1)	1689 (11.4)	864 (11.2)	4779 (9.5)	
2020	7162 (9.8)	1526 (10.3)	884 (11.4)	4752 (9.5)	
2021	7286 (10.0)	1600 (10.8)	975 (12.6)	4711 (9.4)	
Age, y					
18-24	5444 (7.5)	1118 (7.5)	547 (7.1)	3779 (7.5)	<.001
25-29	14 133 (19.4)	2993 (20.2)	1472 (19.0)	9668 (19.2)	
30-34	27 423 (37.7)	5781 (39.0)	2956 (38.2)	18 686 (37.2)	
35-39	20 809 (28.6)	4127 (27.8)	2211 (28.6)	14 471 (28.8)	
≥40	5027 (6.9)	809 (5.5)	546 (7.1)	3672 (7.3)	
Education					
<High school	6021 (8.3)	1492 (10.1)	696 (9.0)	3833 (7.6)	<.001
High school or GED	11 878 (16.3)	2277 (15.4)	1210 (15.6)	8391 (16.7)	
Some college	18 441 (25.3)	3257 (22.0)	1922 (24.9)	13 262 (26.4)	
≥College	34 805 (47.8)	7406 (49.9)	3721 (48.1)	23 678 (47.1)	
Missing	1691 (2.3)	396 (2.7)	183 (2.4)	1112 (2.2)	
Insurance type					
Public, self-pay, or other	36 613 (50.3)	7696 (51.9)	3964 (51.3)	24 953 (49.6)	<.001
Private	36 223 (49.7)	7132 (48.1)	3768 (48.7)	25 323 (50.4)	
Born in the US					
Yes	47 717 (65.5)	9203 (62.1)	4748 (61.4)	33 766 (67.2)	<.001
No	25 119 (34.5)	5625 (37.9)	2984 (38.6)	16 510 (32.8)	
Chronic hypertension					
No	71 296 (97.9)	14 638 (98.7)	7556 (97.7)	49 102 (97.7)	<.001
Yes	1540 (2.1)	190 (1.3)	176 (2.3)	1174 (2.3)	
Preeclampsia					
No	67 039 (92.0)	13 872 (93.6)	6855 (88.7)	46 312 (92.1)	<.001
Yes	5797 (8.0)	956 (6.4)	877 (11.3)	3964 (7.9)	
Gestational diabetes^b^					
No	64 918 (90.7)	13 654 (93.0)	6862 (90.3)	44 402 (90.1)	<.001
Yes	6638 (9.3)	1030 (7.0)	734 (9.7)	4874 (9.9)	
Preexisting diabetes					
No	71 556 (98.2)	14 684 (99.0)	7596 (98.2)	49 276 (98.0)	<.001
Yes	1280 (1.8)	144 (1.0)	136 (1.8)	1000 (2.0)	
Parity					
1 Prior birth	57 285 (78.6)	8958 (60.4)	6317 (81.7)	42 010 (83.6)	<.001
2 Prior births	10 751 (14.8)	3685 (24.9)	965 (12.5)	6101 (12.1)	
≥3 Prior births	4800 (6.6)	2185 (14.7)	450 (5.8)	2165 (4.3)	
Prepregnancy BMI					
<18.5	1529 (2.1)	421 (2.8)	154 (2.0)	954 (1.9)	<.001
18.5-24.9	29 847 (41.0)	7261 (49.0)	3022 (39.1)	19 564 (38.9)	
25.0-29.9	19 682 (27.0)	3810 (25.7)	2181 (28.2)	13 691 (27.2)	
≥30.0	19 219 (26.4)	2807 (18.9)	2117 (27.4)	14 295 (28.4)	
Missing	2559 (3.5)	529 (3.6)	258 (3.3)	1772 (3.5)	
Gestational age, wk					
34-36	4795 (6.6)	1048 (7.1)	505 (6.5)	3242 (6.4)	<.001
37-38	19 096 (26.2)	3881 (26.2)	1941 (25.1)	13 274 (26.4)	
39-40	44 211 (60.7)	8288 (55.9)	4432 (57.3)	31 491 (62.6)	
41	4734 (6.5)	1611 (10.9)	854 (11.0)	2269 (4.5)	
Obstetric comorbidity index score for SMM, mean (SD)					
Excluding transfusion	5.70 (8.72)	4.93 (7.79)	6.81 (9.86)	5.75 (8.78)	<.001
Including transfusion	4.04 (6.13)	3.52 (5.59)	5.05 (6.95)	4.03 (6.13)	<.001
Severe maternal morbidity					
Excluding transfusion					
No	72 283 (99.2)	14 760 (99.5)	7604 (98.3)	49 919 (99.3)	<.001
Yes	553 (0.8)	68 (0.5)	128 (1.7)	357 (0.7)	
Including transfusion					
No	71 484 (98.1)	14 624 (98.6)	7417 (95.9)	49 443 (98.3)	<.001
Yes	1352 (1.9)	204 (1.4)	315 (4.1)	833 (1.7)	

Abbreviations: BMI, body mass index (calculated as weight in kilograms divided by height in meters squared); GED, General Educational Development; LAC, labor after cesarean delivery; SMM, severe maternal morbidity; VBAC, vaginal birth after cesarean delivery.

^a^ Individuals were categorized as other race and ethnicity if they reported not being of Hispanic or Latino/Latina/Latinx ethnicity and reported a race other than Black or White. This included American Indian or Alaska Native, Asian, Pacific Islander, and unreported race.

^b^ Gestational diabetes was calculated among 71 556 individuals without prepregnancy diabetes.

Birth mode varied significantly by all characteristics examined (Table 1). The proportion of individuals with VBAC and unplanned repeat cesarean birth increased over the study period, while planned repeat cesarean birth decreased. The mean (SD) obstetric comorbidity index score was 5.75 (8.78) among individuals with planned repeat cesarean birth, 4.93 (7.79) among those with VBAC, and 6.81 (9.86) among those with unplanned repeat cesarean birth. Obstetric comorbidity index scores were slightly lower for those who had LAC than for those who had planned repeat cesarean (mean [SD] score, 5.57 [5.57 [8.60] vs 5.75 [8.77]; *P* = .01). Black and Latinx individuals had higher obstetric comorbidity index scores than White individuals in each birth mode category (eTable 2 in Supplement 1); however, the magnitude of the difference in scores by race and ethnicity was similar within each birth mode.

Birth mode, LAC, and SMM by race and ethnicity are shown in Table 2. Higher proportions of White individuals had planned repeat cesarean births, while individuals who were Black, Latinx, and other race or ethnicity were disproportionately represented among those with VBAC and unplanned repeat cesarean birth. For example, 1229 Black individuals (15.3%) had unplanned repeat cesarean birth vs 1687 Latinx individuals (11.5%) and 3763 White individuals (9.1%) (*P* < .001). SMM rates were lower among White birthing people than among birthing people from the other racial and ethnic groups (117 Black individuals [1.5%]; 126 Latinx individuals [0.9%]; 234 White individuals [0.6%] 76 individuals with other race or ethnicity [0.9%]; *P* < .001).

**Table 2.** Birth Mode, LAC, and Severe Maternal Morbidity by Race and Ethnicity Among Individuals With a Prior Cesarean Birth, Massachusetts, 2012-2021

Birth mode	No. (%) (N = 72 836)				*P* value
	Black	Hispanic or Latinx	White	Other^a^	
Vaginal birth after cesarean delivery	1869 (23.3)	3223 (22.0)	7614 (18.4)	2122 (24.1)	<.001
Unplanned repeat cesarean delivery	1229 (15.3)	1687 (11.5)	3763 (9.1)	1053 (12.0)	
Planned repeat cesarean delivery	4924 (61.4)	9754 (66.5)	29 973 (72.5)	5625 (63.9)	
LAC					
No	4943 (61.6)	9781 (66.7)	29 982 (72.5)	5635 (64.0)	<.001
Yes	3079 (38.4)	4883 (33.3)	11 368 (27.5)	3165 (36.0)	
SMM					
Excluding transfusion					
No	7905 (98.5)	14 538 (99.1)	41 116 (99.4)	8724 (99.1)	<.001
Yes	117 (1.5)	126 (0.9)	234 (0.6)	76 (0.9)	
Including transfusion					
No	7777 (96.9)	14 336 (97.8)	40 754 (98.6)	8617 (97.9)	<.001
Yes	245 (3.1)	328 (2.2)	596 (1.4)	183 (2.1)	

Abbreviations: LAC, labor after cesarean delivery; SMM, severe maternal morbidity.

^a^ Individuals were categorized as other race and ethnicity if they reported not being of Hispanic or Latino/Latina/Latinx ethnicity and reported a race other than Black or White. This included American Indian or Alaska Native, Asian, Pacific Islander, and unreported race.

In the adjusted multivariate logistic regression model with 3-category birth mode and race and ethnicity as key independent variables (Table 3), Black individuals had significantly higher odds of SMM compared with White individuals (adjusted odds ratio [AOR], 1.60; 95% CI, 1.25-2.05). Individuals with planned repeat cesarean birth had higher odds of SMM compared with those with VBAC (AOR, 1.57; 95% CI, 1.20-2.06), while individuals with unplanned repeat cesarean birth had 3 times the odds of SMM compared with those with VBAC (AOR, 3.05; 95% CI, 2.23-4.18).

**Table 3.** Adjusted Logistic Regression Results for Severe Maternal Morbidity by Race/Ethnicity and Birth Mode, Massachusetts, 2012-2021

Measure	Model 1^a^		Model 2^a^	
	AOR (95% CI)	*P* value	AOR (95% CI)	*P* value
Race/ethnicity				
Black	1.60 (1.25-2.05)	<.001	0.86 (0.47-1.60)	.64
Hispanic or Latinx	1.13 (0.82-1.56)	.47	0.57 (0.28-1.18)	.13
White	1 [Reference]	NA	1 [Reference]	NA
Other^b^	1.19 (0.92-1.53)	.19	0.69 (0.26-1.83)	.46
Birth mode				
VBAC	1 [Reference]	NA	1 [Reference]	NA
Unplanned repeat cesarean	3.05 (2.23-4.18)	<.001	2.43 (1.49-3.94)	<.001
Planned repeat cesarean	1.57 (1.20-2.06)	.001	1.02 (0.78-1.32)	.89
Interaction of race and ethnicity with birth mode				
Black × unplanned repeat cesarean	NA	NA	1.65 (0.74-3.68)	.22
Black × planned repeat cesarean	NA	NA	2.16 (1.13-4.14)	.02
Hispanic or Latinx × unplanned repeat cesarean	NA	NA	1.55 (0.59-4.08)	.38
Hispanic or Latinx × planned repeat cesarean	NA	NA	2.42 (1.13-5.19)	.02
Other race × unplanned repeat cesarean	NA	NA	1.48 (0.44-4.97)	.53
Other race × planned repeat cesarean	NA	NA	2.02 (0.81-5.05)	.13

Abbreviations: AOR, adjusted odds ratio; NA, not applicable; VBAC, vaginal birth after cesarean delivery.

^a^ Models adjusted for age category, education, insurance type, born in the US, gestational age category, parity, prepregnancy body mass index, obstetric comorbidity score, and year. Standard errors were clustered by hospital. Outcome measure of severe maternal morbidity excludes blood transfusion.

^b^ Individuals were categorized as other race and ethnicity if they reported not being of Hispanic or Latino/Latina/Latinx ethnicity and reported a race other than Black or White. This included American Indian or Alaska Native, Asian, Pacific Islander, and unreported race.

We next added an interaction term to the model to test whether the association of birth mode with SMM varied by race and ethnicity (Table 3). These results indicated that the association between birth mode and SMM did vary by race and ethnicity, with statistically significant interaction terms for several comparisons. The Figure presents adjusted probabilities as percentages across categories from this model to aid in interpretation. Among individuals with VBAC, the estimated percentage with SMM was similar across racial and ethnic groups: 0.58% for White individuals, 0.50% for Black individuals, and 0.34% for Latinx individuals. Among individuals with planned repeat cesarean birth, the estimated percentage with SMM was significantly higher for each racial and ethnic group compared with White birthing people: 1.06% for Black individuals and 0.80% for Latinx individuals, vs 0.59% for White individuals. Among individuals with unplanned repeat cesarean birth, the estimated percentage of SMM was not significantly different across racial and ethnic groups. Planned repeat cesarean birth vs VBAC was associated with an increase in the likelihood of SMM of 0.56 (95% CI, 0.21-0.90) percentage points (*P* = .001) among Black birthing people and 0.46 (95% CI, 0.16-0.76) percentage points (*P* = .003) among Latinx birthing people, while among White individuals, the likelihood of SMM did not differ between planned repeat cesarean birth and VBAC (eTable 3 in Supplement 1). Increases in likelihood of SMM associated with unplanned repeat cesarean birth relative to VBAC ranged from 0.75 (95% CI, 0.30-1.21) percentage points among White individuals to 1.35 (95% CI, 0.74-1.97) percentage points among Black individuals, but these differences were not statistically significant.

**Figure.**
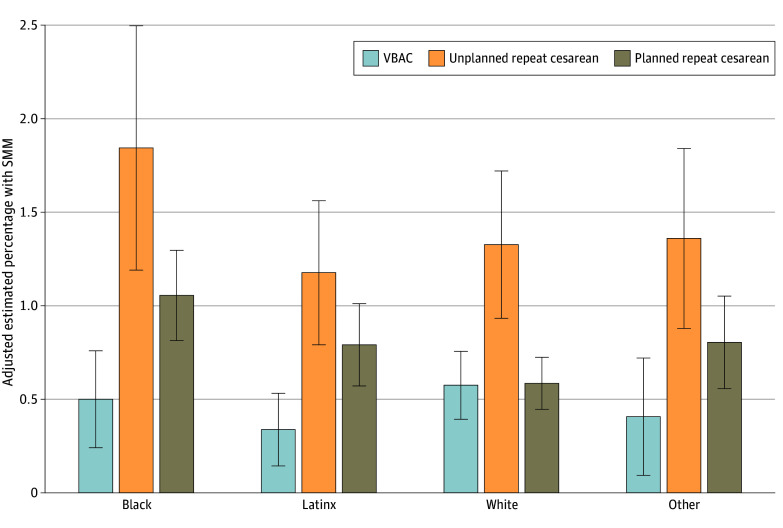
Adjusted Estimated Percentage With Severe Maternal Morbidity (SMM) by Birth Mode and Race and Ethnicity Among Individuals With a Prior Cesarean Delivery, Massachusetts, 2012-2021 Estimates are based on probability of the outcome derived from regression models. Models adjust for age category, education, insurance type, born in the US, gestational age category, prepregnancy body mass index, obstetric comorbidity score, parity, and year. Standard errors were clustered by hospital. SMM excludes blood transfusion. Individuals were categorized as other race or ethnicity if they reported not being of Hispanic or Latino/Latina/Latinx ethnicity and reported a race other than Black or White. This included American Indian or Alaska Native, Asian, Pacific Islander, and unreported race.

In adjusted multivariate logistic regression models with LAC vs planned repeat cesarean birth and race and ethnicity as key risk factors (Table 4), results showed no statistically significant increase in SMM associated with LAC (AOR, 1.16; 95% CI, 0.96-1.41). When an interaction term was added to the model, the main finding for LAC became statistically significant and positive (AOR, 1.52; 95% CI, 1.25-1.83), and the association significantly differed for Latinx and other race and ethnicity birthing people compared with White birthing people. Estimated percentages with SMM by LAC and race and ethnicity from the interaction model are shown in eFigure 2 in Supplement 1, and average marginal effects are presented in eTable 4 in Supplement 1. LAC was associated with a positive and significant average marginal effect on SMM for White birthing people only.

**Table 4.** Adjusted Logistic Regression Results for Severe Maternal Morbidity by Race and Ethnicity and LAC, Massachusetts, 2012-2021

Measure	Model 1^a^		Model 2^a^	
	AOR (95% CI)	*P* value	AOR (95% CI)	*P* value
Race and ethnicity				
Black	1.65 (1.28-2.13)	<.001	1.86 (1.40-2.47)	<.001
Hispanic or Latinx	1.15 (0.83-1.58)	.40	1.39 (0.91-2.12)	.13
White	1 [Reference]	NA	1 [Reference]	NA
Other^b^	1.19 (0.93-1.53)	.17	1.41 (1.10-1.81)	.007
LAC				
No	1 [Reference]	NA	1 [Reference]	NA
Yes	1.16 (0.96-1.41)	.13	1.52 (1.25-1.83)	<.001
Interaction of race and ethnicity with LAC				
Black × LAC	NA	NA	0.71 (0.45-1.13)	.15
Hispanic or Latinx × LAC	NA	NA	0.57 (0.36-0.90)	.02
Other race × LAC	NA	NA	0.63 (0.41-0.96)	.03

Abbreviations: AOR, adjusted odds ratio; LAC, labor after cesarean delivery; NA, not applicable.

^a^ Models adjusted for age category, education, insurance type, born in the US, gestational age category, parity, prepregnancy body mass index, obstetric comorbidity score, and year. Standard errors were clustered by hospital. Outcome measure of severe maternal morbidity excludes blood transfusion.

^b^ Individuals were categorized as other race and ethnicity if they reported not being of Hispanic or Latino/Latina/Latinx ethnicity and reported a race other than Black or White. This included American Indian or Alaska Native, Asian, Pacific Islander, and unreported race.

In sensitivity analyses using the SMM measure including blood transfusion, results were generally consistent in direction and magnitude (eTable 5 and eTable 6 in Supplement 1). However, in this analysis, there was also variation in the magnitude of the increase in SMM associated with unplanned repeat cesarean birth by race and ethnicity, with larger increases in SMM for Black birthing people with unplanned cesarean birth vs VBAC than for White birthing people. Additionally, there was racial and ethnic variation in the association between LAC and SMM: for White birthing people, LAC was associated with an increase in SMM, while for Latinx birthing people, LAC was not associated with an increase in SMM. Average marginal effects from these models are presented in eTable 7 and eTable 8 in Supplement 1.

## Discussion

In this cross-sectional study, we found that SMM rates for Black and White birthing people with VBAC were not different from one another. However, among Black and Latinx birthing people, rates of SMM were higher for planned cesarean birth relative to VBAC, while among White birthing people, SMM rates for planned repeat cesarean birth and VBAC were similar. These results were consistent in a sensitivity analysis including blood transfusion in the SMM measure. In our main analysis, Black birthing people with unplanned repeat cesarean delivery had the highest estimated likelihood of SMM of the racial and ethnic groups examined, but the increase in the chance of SMM associated with unplanned repeat cesarean birth relative to VBAC or planned repeat cesarean birth was not statistically different across racial and ethnic groups.

Our results were consistent with research from the 1990s and early 2000s demonstrating lower rates of maternal morbidity with VBAC compared with planned repeat cesarean delivery and higher rates of maternal morbidity with unplanned repeat cesarean delivery, although these older studies measured specific morbidity categories rather than a composite and included conditions that were not part of our SMM measure.^28,29^ However, our findings revealed important differences in the patterns of SMM by birth mode when disaggregated by race and ethnicity among individuals with a prior cesarean delivery. Notably, Black individuals with VBAC had equivalent SMM rates to White individuals with VBAC. Additionally, for White birthing people, SMM rates were not significantly different between VBAC and planned repeat cesarean delivery, whereas for Black and Latinx birthing people, SMM was higher with planned repeat cesarean delivery relative to VBAC.

More recent studies from other countries comparing maternal morbidity by LAC vs planned repeat cesarean birth have had mixed results, with one study showing no difference,^30^ and another finding higher maternal morbidity rates among those with LAC.^31^ A study of LAC among individuals with 2 prior cesarean deliveries in California found no difference in SMM by LAC.^22^ In adjusted analyses, we found no association between LAC and SMM. However, when we examined potential interaction between LAC and race and ethnicity, we found that the association between LAC and SMM was driven by White birthing people; this should be confirmed in larger samples.

An individual’s likelihood of achieving VBAC may be an important consideration in the decision to have LAC, given the higher rates of morbidity associated with unplanned cesarean birth; thus, there has been substantial interest in tools to provide this information. The Maternal-Fetal Units Network’s VBAC calculator was a commonly used prognostic tool during the time of our study.^32^ The calculator generated an estimated probability of VBAC success based on factors that could be known prior to labor, including Black race and Hispanic ethnicity, either of which resulted in a lower estimated probability of success. Criticisms of the inclusion of race and ethnicity in this calculator highlighted the inappropriateness of including socially constructed variables as biological risk factors and noted that their inclusion could exacerbate disparities,^33,34^ leading to development of a new version of the calculator without race or ethnicity.^35,36^ However, during the time of our study, clinicians would only have had access to the calculator that included race and ethnicity. As such, clinicians may have been less likely to offer or encourage LAC among Black or Hispanic patients and potentially more likely to recommend an unplanned cesarean delivery to these groups during labor.

Our findings suggest a need to understand and address worse surgical outcomes for birthing people from minoritized racial and ethnic groups, such as Black and Latinx individuals, with a prior cesarean delivery, especially those who have a planned repeat cesarean delivery. Race and ethnicity is a social construct and not a biological characteristic; changes to clinical care are critical in addressing manifestations of structural and interpersonal racism within the health care system and improving outcomes.^4,37,38,39^ While we were not able to directly examine the mechanisms underlying our results, it is possible that due to structural racism and residential segregation, Black and Latinx birthing people are more concentrated in lower-resourced hospitals that provide lower-quality care.^4^ Future studies should explore the contribution of hospital characteristics and resources to racial and ethnic disparities in SMM for those with a prior cesarean delivery. A recent multicenter trial in Quebec, Canada, randomized hospitals to receive a multifaceted intervention to support patient decision-making around LAC and to promote guideline-concordant intrapartum care for individuals with a prior cesarean delivery. This intervention successfully reduced serious maternal and perinatal morbidity, while rates of LAC remained unchanged.^40^ Similar practices implemented in US hospital settings may have the potential to improve outcomes for individuals with a prior cesarean birth and to reduce racial and ethnic disparities through the standardization of care that is aligned with best practices.

### Strengths and Limitations

Strengths of this study include use of a statewide database, use of birth certificate data to ascertain race and ethnicity (based on self-report), measurement of SMM based on the Centers for Disease Control and Prevention definition, and adjustment for obstetric risk with a validated measure. However, there were also limitations to our study. First, in some individuals, the condition that was an indicator of SMM may also have been the reason for the unplanned cesarean delivery. Thus, birth mode cannot be interpreted as causing SMM. Second, an individual planning to have a repeat cesarean birth who went into labor would be classified as having unplanned repeat cesarean birth, even though they did not intend to have LAC; as a result, there may be some misclassification of birth mode. Third, due to lifetime exposure to racism, Black individuals may enter pregnancy in worse health in ways that impact SMM from planned cesarean birth but that we were not able to measure. However, our analysis accounted for a comorbidity index specifically designed to capture SMM risk. Fourth, the birthing population in Massachusetts differs from the national birthing population, including in racial and ethnic composition. However, Massachusetts trends in SMM and VBAC rates are similar to those seen nationally.^41,42^ Fifth, there is substantial variation in lived experience within the racial and ethnic categories used in our analysis, with potential differences by region or country of origin and English language proficiency, among other factors, which influence health care and birth experiences. This should be further explored in future research. Sixth, our analysis relied on administrative and vital statistics data, which may not capture all relevant clinical risk factors. However, combining these data sources enhances the validity of available measures and enables analysis of a broad population.

## Conclusions

In this cross-sectional study of individuals with a history of cesarean delivery, SMM rates by birth mode differed by race and ethnicity. Black and Latinx birthing people experienced higher rates of SMM with planned repeat cesarean birth compared with VBAC, while among White birthing people, SMM was similar for planned repeat cesarean birth and VBAC. Identifying factors contributing to higher SMM rates among Black and Latinx birthing people with planned repeat cesarean delivery is necessary to improve care quality and to promote equity.

## Notes

Supplement 1. **eFigure 1. **Sample Restriction Criteria **eFigure 2.** Adjusted Predicted Percentage With Severe Maternal Morbidity by Labor After Cesarean and race/Ethnicity Among Individuals With a Prior Cesarean **eTable 1.** *ICD* Codes Used to Identify Labor After Cesarean **eTable 2.** Mean Obstetric Comorbidity Scores by Birth Mode and Race/Ethnicity **eTable 3.** Average Marginal Effect of Birth Mode on Severe Maternal Morbidity by Race/Ethnicity **eTable 4.** Average Marginal Effect of Labor After Cesarean on Severe Maternal Morbidity by Race/Ethnicity **eTable 5.** Adjusted Logistic Regression Results for Severe Maternal Morbidity Including Blood Transfusion by Race/Ethnicity and Birth Mode, Massachusetts, 2012-2021 **eTable 6.** Adjusted Logistic Regression Results for Severe Maternal Morbidity Including Blood Transfusion by Race/Ethnicity and Labor After Cesarean, Massachusetts, 2019-2021 **eTable 7.** Average Marginal Effect of Birth Mode Category on Severe Maternal Morbidity by Race/Ethnicity **eTable 8.** Average Marginal Effect of Labor After Cesarean on Severe Maternal Morbidity by Race/Ethnicity

Supplement 2.
Data Sharing Statement
