# Supplementary material for: Severe Maternal Morbidity by Race and Ethnicity and Birth Mode Among Individuals With a Prior Cesarean Birth
Source: JAMA Netw Open. 2025 Jun 3;8(6):e2513578. doi: 10.1001/jamanetworkopen.2025.13578 (PMC12134949; doi:10.1001/jamanetworkopen.2025.13578)
Supplement: Supplement 1. — eFigure 1. Sample Restriction Criteria eFigure 2. Adjusted Predicted Percentage With Severe Maternal Morbidity by Labor After Cesarean and race/Ethnicity Among Individuals With a Prior Cesarean eTable 1. ICD Codes Used to Identify Labor After Cesarean eTable 2. Mean Obstetric Comorbidity Scores by Birth Mode and Race/Ethnicity eTable 3. Average Marginal Effect of Birth Mode on Severe Maternal Morbidity by Race/Ethnicity eTable 4. Average Marginal Effect of Labor After Cesarean on Severe Maternal Morbidity by Race/Ethnicity eTable 5. Adjusted Logistic Regression Results for Severe Maternal Morbidity Including Blood Transfusion by Race/Ethnicity and Birth Mode, Massachusetts, 2012-2021 eTable 6. Adjusted Logistic Regression Results for Severe Maternal Morbidity Including Blood Transfusion by Race/Ethnicity and Labor After Cesarean, Massachusetts, 2019-2021 eTable 7. Average Marginal Effect of Birth Mode Category on Severe Maternal Morbidity by Race/Ethnicity eTable 8. Average Marginal Effect of Labor After Cesarean on Severe Maternal Morbidity by Race/Ethnicity [file jamanetwopen-e2513578-s001.pdf]

Supplemental Online Content

Attanasio LB, Goff S, Hardeman R, Laws H, Srinivas S. Severe maternal morbidity by race and ethnicity and birth mode among individuals with a prior cesarean birth. *JAMA Netw Open*. 2025;8(6):e2513578. doi:10.1001/jamanetworkopen.2025.13578

eFigure 1. Sample Restriction Criteria

eFigure 2. Adjusted Predicted Percentage With Severe Maternal Morbidity by Labor After Cesarean and race/Ethnicity Among Individuals With a Prior Cesarean

eTable 1. *ICD* Codes Used to Identify Labor After Cesarean

eTable 2. Mean Obstetric Comorbidity Scores by Birth Mode and Race/Ethnicity

eTable 3. Average Marginal Effect of Birth Mode on Severe Maternal Morbidity by Race/Ethnicity

eTable 4. Average Marginal Effect of Labor After Cesarean on Severe Maternal Morbidity by Race/Ethnicity

eTable 5. Adjusted Logistic Regression Results for Severe Maternal Morbidity Including Blood Transfusion by Race/Ethnicity and Birth Mode, Massachusetts, 2012-2021

eTable 6. Adjusted Logistic Regression Results for Severe Maternal Morbidity Including Blood Transfusion by Race/Ethnicity and Labor After Cesarean, Massachusetts, 2019-2021

eTable 7. Average Marginal Effect of Birth Mode Category on Severe Maternal Morbidity by Race/Ethnicity

eTable 8. Average Marginal Effect of Labor After Cesarean on Severe Maternal Morbidity by Race/Ethnicity

This supplemental material has been provided by the authors to give readers additional information about their work.

© 2025 Attanasio LB et al. JAMA Network Open.

eFigure 1. Sample Restriction Criteria

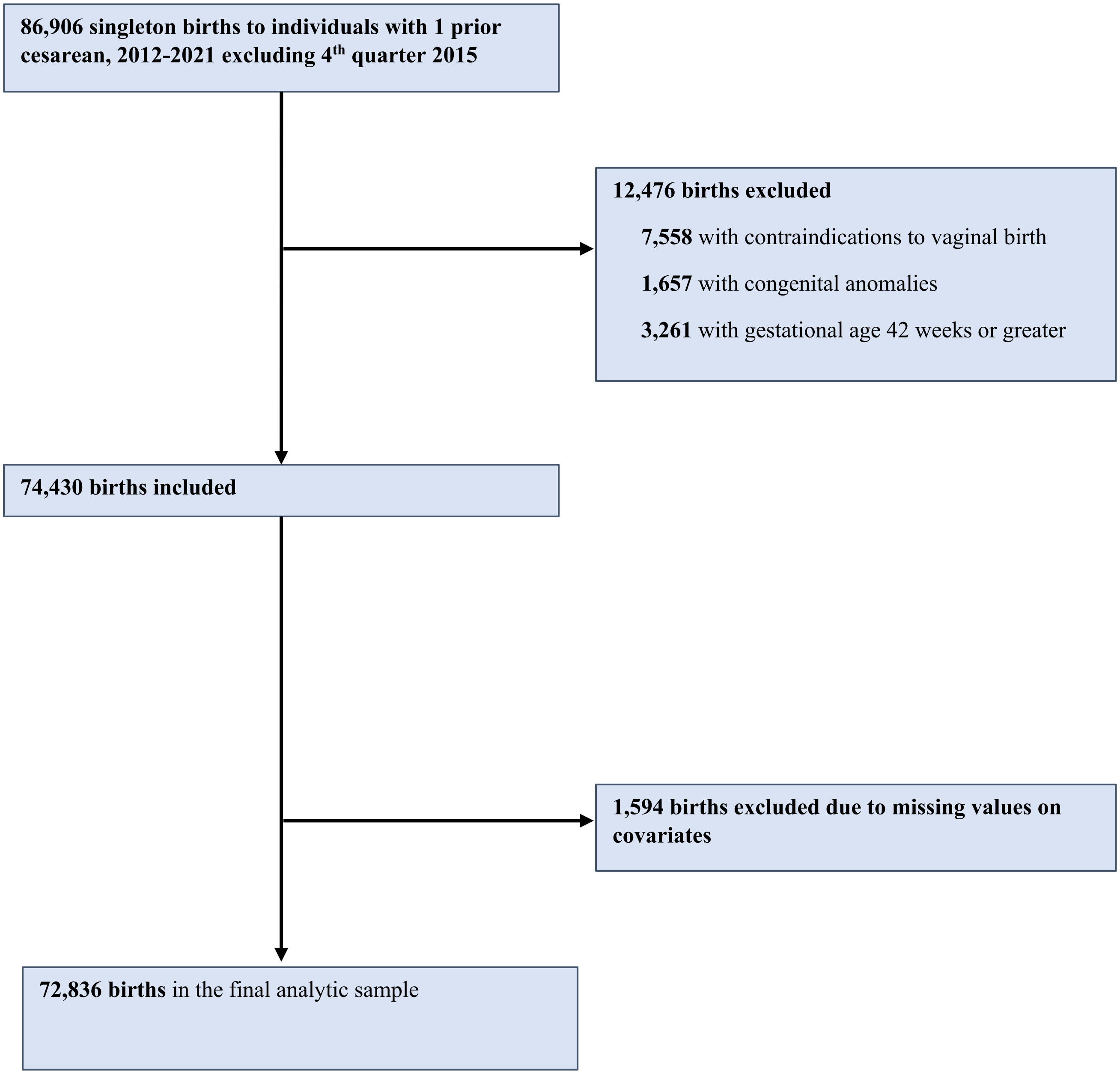

© 2025 Attanasio LB et al. JAMA Network Open.

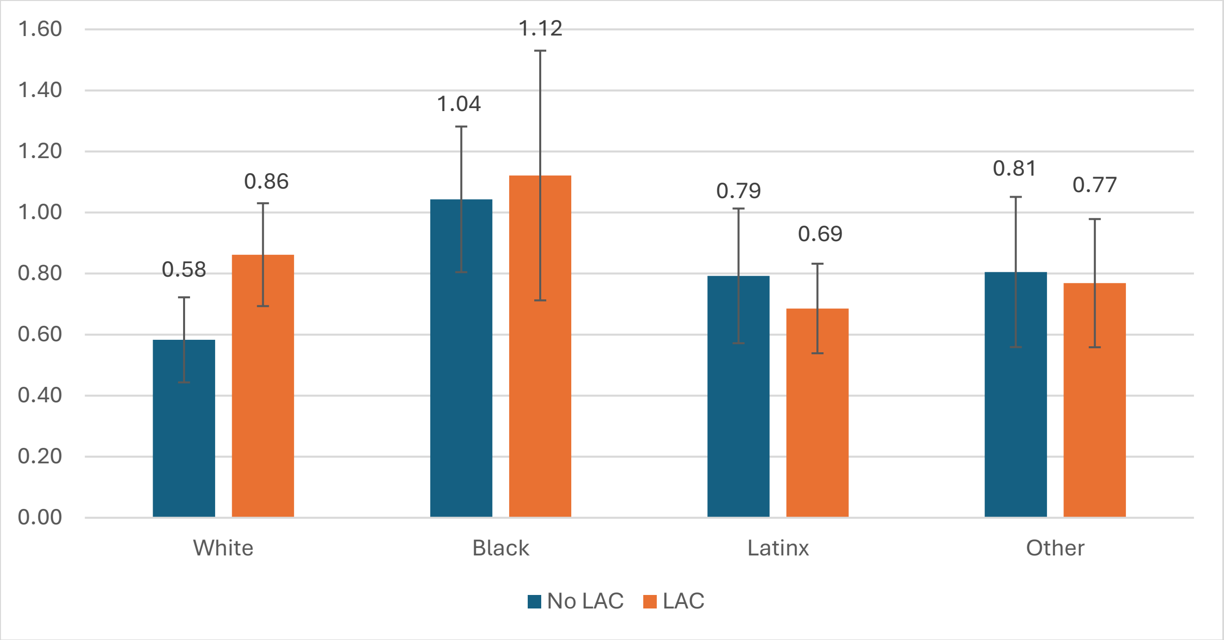

eFigure 2. Adjusted predicted percentage with severe maternal morbidity by labor after cesarean and race/ethnicity among individuals with a prior cesarean. Estimates are predicted probability of the outcome derived from regression models. Models adjust for age category, education, insurance type, born in the US, gestational age category, pre-pregnancy body mass index, obstetric comorbidity score, parity, and year. Standard errors were clustered by hospital. Outcome measure of severe maternal morbidity excludes blood transfusion. Individuals were categorized as "Other" race/ethnicity if they reported not being of Hispanic/Latino/a/x ethnicity and reported a race other than White or Black. This included Asian, Pacific Islander, American Indian/Alaska Native, and unreported race.

© 2025 Attanasio LB et al. JAMA Network Open.

**eTable 1. ICD codes used to identify labor after cesarean**	
**ICD-9**	**ICD-10**
Any one of these codes:	
72.0, 72.29, 72.4, 72.71, 73.01, 73.1,73.3, 73.4, 73.51, 73.59, 73.6, 75.62	10D07Z3, 10D07Z4, 10S07ZZ, 0W8NXZZ , 10D0726, 10900ZC, 10907ZC, 0U7C7ZZ, 10J07ZZ, 3E033VJ, 0U7C7DZ, 0U7C7ZZ, 3E0DXGC, 3E0P7GC, 10E0XZZ, 10E0XZZ, 0W8NXZZ, 0DQP7ZZ, 0DQP8ZZ, 0DQQ0ZZ, 0DQQ3ZZ, 0DQQ4ZZ, 0DQQ7ZZ, 0DQQ8ZZ, 0DQR0ZZ, 0DQR3ZZ, 0DQR4ZZ
Any two of these codes:	
650, 659.01, 659.11, 660.21, 660.31, 660.41, 660.61, 660.71, 660.81, 660.91, 661.01, 661.11, 661.21, 662.01, 662.11, 662.21, 664.01, 664.11, 664.21, 664.31, 664.51, 664.61, 664.81, 665.11, 669.51	O80, O611, O610, O655, O660, O661, O665, O668, O669, O620, O621, O622, O630, O639, O631, O700, O701, O7020, O703, O717, O704, O7182, O711, O665

© 2025 Attanasio LB et al. JAMA Network Open.

eTable 2. Mean obstetric comorbidity scores by birth mode and race/ethnicity.

	White	Black	Latinx	Other[a]	Black-White difference	Latinx-White difference
VBAC						
Obstetric comorbidity index score for SMM excluding transfusion	3.29	6.63	5.55	4.38	3.34	2.26
Obstetric comorbidity index score for SMM including transfusion	2.97	5.07	4.18	3.12	2.10	1.21
Unplanned repeat cesarean						
Obstetric comorbidity index score for SMM excluding transfusion	6.05	8.72	7.81	5.70	2.67	1.76
Obstetric comorbidity index score for SMM including transfusion	4.33	6.77	5.91	4.22	2.44	1.58
Planned repeat cesarean						
Obstetric comorbidity index score for SMM excluding transfusion	5.38	8.26	6.11	4.86	2.88	0.73
Obstetric comorbidity index score for SMM including transfusion	3.62	6.15	4.56	3.44	2.53	0.94

[a]Individuals were categorized as "Other" race/ethnicity if they reported not being of Hispanic/Latino/a/x ethnicity and reported a race other than White or Black. This included Asian, Pacific Islander, American Indian/Alaska Native, and unreported race.

© 2025 Attanasio LB et al. JAMA Network Open.

eTable 3. Average marginal effect of birth mode on severe maternal morbidity by race/ethnicity.

	AME	p
Relative to VBAC		
White		
Unplanned repeat cesarean	0.76	0.001
Planned repeat cesarean	0.01	0.89
Black		
Unplanned repeat cesarean	1.35	<0.001
Planned repeat cesarean	0.56	0.001
Latinx		
Unplanned repeat cesarean	0.84	0.001
Planned repeat cesarean	0.46	0.003
Other		
Unplanned repeat cesarean	0.96	0.005
Planned repeat cesarean	0.40	0.03
Relative to planned repeat cesarean		
White		
Unplanned repeat cesarean	0.75	<0.001
VBAC	-0.01	0.89
Black		
Unplanned repeat cesarean	0.79	0.03
VBAC	-0.56	0.001
Latinx		
Unplanned repeat cesarean	0.39	0.11
VBAC	-0.46	0.003
Other		
Unplanned repeat cesarean	0.56	0.07
VBAC	-0.40	0.03

Abbreviations: AME, average marginal effect; VBAC, vaginal birth after cesarean

Individuals were categorized as "Other" race/ethnicity if they reported not being of Hispanic/Latino/a/x ethnicity and reported a race other than White or Black. This included Asian, Pacific Islander, American Indian/Alaska Native, and unreported race.

© 2025 Attanasio LB et al. JAMA Network Open.

eTable 4. Average marginal effect of labor after cesarean on severe maternal morbidity by race/ethnicity.

	AME	p
White	0.28	<0.001
Black	0.08	0.75
Latinx	-0.11	0.46
Other	-0.04	0.84

Abbreviations: AME, average marginal effect
Outcome is severe maternal morbidity excluding blood transfusion.

[a]Individuals were categorized as "Other" race/ethnicity if they reported not being of Hispanic/Latino/a/x ethnicity and reported a race other than White or Black. This included Asian, Pacific Islander, American Indian/Alaska Native, and unreported race.

© 2025 Attanasio LB et al. JAMA Network Open.

eTable 5. Adjusted logistic regression results for severe maternal morbidity including blood transfusion by race/ethnicity and birth mode, Massachusetts, 2012-2021.

	Model 1			Model 2		
	**AOR**	**95% CI**	**p**	**AOR**	**95% CI**	**p**
Race and ethnicity						
White	1.00	--	--	1.00	--	--
Black	1.58	(1.35-1.85)	<0.001	0.86	(0.61-1.21)	0.39
Latinx	1.32	(1.07-1.64)	0.01	0.74	(0.51-1.09)	0.13
Other[a]	1.34	(1.09-1.65)	0.005	1.08	(0.77-1.52)	0.66
Birth mode						
VBAC	1.00	--	--	1.00	--	--
Unplanned repeat cesarean	2.60	(1.99-3.40)	<0.001	2.06	(1.4-3.02)	<0.001
Planned repeat cesarean	1.22	(0.98-1.53)	0.07	0.88	(0.7-1.12)	0.30
Race/ethnicity*birth mode						
Latinx*Unplanned repeat cesarean				1.60	(0.94-2.74)	0.08
Latinx*Planned repeat cesarean				2.09	(1.39-3.13)	<0.001
Black*Unplanned repeat cesarean				1.77	(1.14-2.76)	0.01
Black*Planned repeat cesarean				2.15	(1.43-3.23)	<0.001
Other race*Unplanned repeat cesarean				1.33	(0.79-2.24)	0.28
Other race*Planned repeat cesarean				1.28	(0.82-2.00)	0.28

Abbreviations: AOR, adjusted odds ratio; 95% CI, 95% confidence interval; VBAC, vaginal birth after cesarean

Models adjusted for age category, education, insurance type, born in the US, gestational age category, parity, pre-pregnancy body mass index, obstetric comorbidity score, and year. Standard errors were clustered by hospital.

Individuals were categorized as "Other" race/ethnicity if they reported not being of Hispanic/Latino/a/x ethnicity and reported a race other than White or Black. This included Asian, Pacific Islander, American Indian/Alaska Native, and unreported race.

© 2025 Attanasio LB et al. JAMA Network Open.

eTable 6. Adjusted logistic regression results for severe maternal morbidity including blood transfusion by race/ethnicity and labor after cesarean, Massachusetts, 2019-2021.

	Model 1			Model 2		
	**AOR**	**95% CI**	**p**	**AOR**	**95% CI**	**p**
Race and ethnicity						
White	1.00	--	--	1.00	--	--
Black	1.63	(1.39-1.91)	<0.001	1.84	(1.49-2.27)	<0.001
Hispanic or Latinx	1.34	(1.09-1.66)	0.007	1.57	(1.17-2.09)	0.002
Other	1.35	(1.09-1.66)	0.006	1.39	(1.10-1.76)	0.007
LAC						
No	1.00	--	--	1.00	--	--
Yes	1.33	(1.17-1.52)	<0.001	1.59	(1.36-1.87)	<0.001
Race/ethnicity*LAC						
Latinx*LAC				0.65	(0.46-0.92)	0.01
Black*LAC				0.73	(0.51-1.03)	0.07
Other race*LAC				0.91	(0.68-1.22)	0.52

Abbreviations: AOR, adjusted odds ratio; 95% CI, 95% confidence interval; LAC, labor after cesarean

Models adjusted for age category, education, insurance type, born in the US, gestational age category, parity, pre-pregnancy body mass index, obstetric comorbidity score, and year. Standard errors were clustered by hospital. Outcome measure of severe maternal morbidity includes blood transfusion.

Individuals were categorized as "Other" race/ethnicity if they reported not being of Hispanic/Latino/a/x ethnicity and reported a race other than White or Black. This included Asian, Pacific Islander, American Indian/Alaska Native, and unreported race.

© 2025 Attanasio LB et al. JAMA Network Open.

eTable 7. Average marginal effect of birth mode category on severe maternal morbidity by race/ethnicity.

	AME	p
Relative to VBAC		
White		
Unplanned repeat cesarean	1.49	0.001
Planned repeat cesarean	-0.17	0.33
Black		
Unplanned repeat cesarean	3.13	<0.001
Planned repeat cesarean	1.10	<0.001
Latinx		
Unplanned repeat cesarean	2.40	<0.001
Planned repeat cesarean	0.91	0.001
Other[a]		
Unplanned repeat cesarean	2.57	<0.001
Planned repeat cesarean	0.20	0.60
Relative to planned repeat cesarean		
White		
Unplanned repeat cesarean	1.66	<0.001
VBAC	0.17	0.33
Black		
Unplanned repeat cesarean	2.03	<0.001
VBAC	-1.10	<0.001
Latinx		
Unplanned repeat cesarean	1.49	0.004
VBAC	-0.91	0.001
Other		
Unplanned repeat cesarean	2.37	<0.001
VBAC	-0.20	0.60

© 2025 Attanasio LB et al. JAMA Network Open.

Abbreviations: AME, average marginal effect; VBAC, vaginal birth after cesarean

Outcome is severe maternal morbidity including blood transfusion.

Individuals were categorized as "Other" race/ethnicity if they reported not being of Hispanic/Latino/a/x ethnicity and reported a race other than White or Black. This included Asian, Pacific Islander, American Indian/Alaska Native, and unreported race.

© 2025 Attanasio LB et al. JAMA Network Open.

eTable 8. Average marginal effect of labor after cesarean on severe maternal morbidity by race/ethnicity.

	AME	p
White	0.74	<0.001
Black	0.35	0.357
Latinx	0.06	0.818
Other	0.76	0.035

Abbreviations: AME, average marginal effect

Outcome is severe maternal morbidity including blood transfusion.

Individuals were categorized as "Other" race/ethnicity if they reported not being of Hispanic/Latino/a/x ethnicity and reported a race other than White or Black. This included Asian, Pacific Islander, American Indian/Alaska Native, and unreported race.

© 2025 Attanasio LB et al. JAMA Network Open.
