# Supplementary material for: Severe Maternal Morbidity by Race and Ethnicity and Birth Mode Among Individuals With a Prior Cesarean Birth
Source: JAMA Netw Open. 2025 Jun 3;8(6):e2513578. doi: 10.1001/jamanetworkopen.2025.13578 (PMC12134949; doi:10.1001/jamanetworkopen.2025.13578)
Supplement: Supplement 2. — Data Sharing Statement [file jamanetwopen-e2513578-s002.pdf]

Data Sharing Statement

Attanasio. Severe Maternal Morbidity by Race and Ethnicity and Birth Mode Among Individuals With a Prior Cesarean Birth. *JAMA Netw Open*. Published June 03, 2025. doi:10.1001/jamanetworkopen.2025.13578

Data
Data available: No

Additional Information
Explanation for why data not available: The Pregnancy to Early Life Longitudinal Data System (PELL) is disseminated by the Massachusetts Department of Public Health and the Center for Health Information and Analysis. The authors do not have permission to share the data.
